# Straightening a Limb by Dr. Brainard’s Method

**Published:** 1859-03

**Authors:** Paul F. Eve


(From the Nashville Medical Journal for March, 1859.)

*Straightening a Limb by Dr. Brainard's Method. By Paul F. Eve, M.D.*

During the past month (January), I have succeeded by the operation proposed by Professor Daniel Brainard, M.D., of the Rush Medical College, Chicago, Illinois, in restoring a leg to the straight position. It was performed before our class.

The patient is of Sequachey Valley, East Tennessee, now ten years old, who six years ago sustained a fracture of both bones of his left leg; then a second injury at the site of the first, and the case was subsequently neglected. There had resulted a permanent osseous union, but with the lower third of the limb bent an angle of about sixty degrees from the straight line. The tibia and fibula were both flattened latterly, and the boy used a pair of crutches for locomotion.

Placed under chloroform and ether, with a brad awl the tibia and fibula were several times perforated through one opening made in the skin, and the bones re-fractured by the hands. The larger bone readily yielded, but the smaller one was not sufficiently divided. Owing to the severity of the operation, and the free use of the anæsthetic agents, we desisted for the time. Ten days afterwards the fibula was again attacked by the awl, and then the limb brought perfectly straight. It was now placed in a fracture box, extension and counter-extension maintained as usual, and a weight—a pound of shot in a bag—retained over the point where the angle existed, so as to assist in keeping it straight. The leg not having been developed, is about two inches shorter than its corresponding fellow. The patient has returned home.

The case is not presented as a cure, but to exhibit how easily it was brought into a straight position by an operation, devised by an American, honored by our National Medical Association for it in 1854, and which is so simple when compared with former operative procedures on the bones for deformities.

